# Using Telemedicine to Provide Education for the Symptomatic Patient with Chronic Respiratory Disease

**DOI:** 10.3390/life11121317

**Published:** 2021-11-29

**Authors:** Felicity C. Blackstock, Nicola J. Roberts

**Affiliations:** 1 Physiotherapy, School of Health Sciences, Western Sydney University, Penrith, NSW 2751, Australia; 2 Physiotherapy, School of Allied Health, Exercise and Sports Sciences, Charles Sturt University, Bathurst, NSW 2795, Australia; 3 Nursing and Community Health, School of Health and Life Sciences, Glasgow Caledonia University, Glasgow G4 0BA, UK; nicola.roberts@gcu.ac.uk

**Keywords:** technology-enabled patient education, applications education, web-based education, chronic respiratory conditions

## Abstract

Technology-enabled learning, using computers, smartphones, and tablets, to educate patients on their respiratory disease and management has grown over the last decade. This shift has been accelerated by the global COVID-19 pandemic and the need to socially distance for public health. Thirteen recently published papers examined experience, knowledge, skills and attitude acquisition, behaviour change, and impact on health outcomes of patient education using technology (websites and mobile device applications) for people with chronic respiratory disease. Technology-enabled patient education that includes relevant information, with activities that encourage the patient to interact with the digital platform, appears to lead to better patient experience and may increase learning and behaviour change with improved quality of life. Developing online relationships with healthcare providers, lower digital capabilities, and poor access to a computer/smartphone/tablet, appear to be barriers that need to be overcome for equity in access. Maintaining the principles of quality educational design, ensuring interactive experiences for patient involvement in the educational activities, patient co-design, healthcare professionals connecting with experts in the field of technology-enabled learning for development of education models, and ongoing research lead to the best patient outcomes in technology-enabled education for respiratory disease.

## 1. Introduction

Technology-enabled learning, using computers, smart phones, and tablets, to support patients to understand their respiratory disease and management approaches has been growing over the last decade. Initially, this was through websites and online videos platforms, such as YouTube [1], but in more recent times, telehealth has opened new avenues for supporting chronic respiratory patients with learning about their disease for self-management. A 2019 audit of the two digital distribution platforms (Apple App Store and Google Play Store) by Sleurs et al. (2019) identified 112 apps for supporting people to manage their chronic respiratory condition using a smartphone or tablet device [2]. These applications have moved from static information provision to being interactive platforms that can drive communication between patient, healthcare provider(s), and peers. This rapid shift in the creation of technology-enabled patient education and healthcare interactions has been further accelerated by the global COVID-19 pandemic and the need to socially distance for virus transmission minimisation. Healthcare providers and app developers have been prompted to innovate, and technology has been the key to healthcare providers remaining connected with their patients, with patient education being no exception to these new challenges and opportunities.

Patient education and learning is well established as a core component of care for people with chronic respiratory disease [3]. Learning is considered to be the acquisition of new knowledge, practical skills, and attitudes, and in the context of healthcare, this is usually to support or educate a person to become more independent with the management of their health condition. Learning underpins the end goal of a person with a health condition changing their everyday activities and behaviours for successful health outcomes. This is often termed patient self-management. Specifically, patient education is the activity of supporting a person with chronic respiratory disease to learn information and psychomotor skills and shift their attitudes toward living a healthier life such as regular exercise, monitoring of symptoms, and adherence with medication routines. Patient education can be in the form of a formal structured activity or informal patient encounter, which leads to a conversation or coaching around a specific aspect of the patient’s condition and/or management.

In using technology for patient education, there are two main approaches: synchronous and asynchronous. Synchronous “learning and teaching” occurs when the patient is online at the same time as the healthcare provider and/or their peers and interactive discussions around a topic takes place, for example live sessions online with healthcare providers using video conferencing. Asynchronous “learning and teaching” occurs when the patient accesses learning materials available to them online at a time convenient to them. This is the most common method of learning online for patients through websites and mobile apps on smart devices such as smart phones and tablets. Static material is usually presented in the form of written information and/or videos, with accompanying interactive tasks such as uploading self-monitoring data or completing quizzes or health questionnaires to understand impact of condition on health status [2]. Asynchronous learning can also be in the form of written communications for discussion and application of knowledge that the patient accesses at a convenient time to them.

## 2. Impact of Technology-Enabled Patient Education

The literature evaluating the impact of technology-enabled education for chronic respiratory disease remains sparse but is slowly expanding. However, much of the research evaluating telehealth education for people with chronic respiratory conditions is in the context of pulmonary rehabilitation, where education is delivered in combination with exercise training. When technology-enabled patient education is combined with exercise training in telehealth pulmonary rehabilitation, there is no significant difference in health outcomes of exercise capacity and quality of life compared to face-to-face pulmonary rehabilitation, demonstrating telehealth to be a viable alternative for our post-pandemic world [4]. However, when delivered together with supervised exercise training, it is difficult to isolate the impact of the technology-enabled patient education component specifically. 

In the last 10 years, 12 studies and 1 systematic review examining technology-enabled patient education for people with chronic respiratory disease, identified through a search of CINAHL, evaluated outcomes in isolation from a structured pulmonary rehabilitation (Table 1). Specifically, participants in these studies were provided with a technology platform to access information and activities that develop new knowledge, skills, and attitudes for self-management of chronic respiratory disease (Table 1). The platforms have multiple forms, i.e., websites [5,6,7,8,9,10,11], apps [12,13,14], “serious games” [15], or virtual reality environments [16], incorporating activities for learning information and skills that can be applied to managing their condition. 

As is observed with face-to-face patient education [17,18], a very broad range of approaches has been used across a broad range of learning outcomes and measured with a broad range of outcomes. This makes the interpretation of the literature a challenge. Nonetheless, using Kirkpatrick’s model of evaluation of learning and education, four key levels of impact for participants are presented: user satisfaction with the technology-enabled patient education (level 1), gain of skills and knowledge (level 2), behavioural change following completing the learning activities (level 3), and improved health outcomes (level 4) [19].

**Table 1:** Summary of the literature examining technology-enabled patient education for people with chronic respiratory conditions.

	Respiratory Condition and Sample	Intervention	Evaluation Level(s) * [19]	Outcomes	Challenges; Engagement/Retention
Bourne et al. (2020) [5]; United Kingdom Nested qualitative study as part of a feasibility study (RCT comparing web-based rehabilitation with standard pulmonary rehabilitation)	103 Adults with COPD (MRC 2–5) were randomised (51 web-based PR; 52 standard pulmonary rehabilitation) 20 participants (15 completers and 5 non-completers were interviewed)	Website programme based of the SPACE for COPD self-management manual. Four stages consisting of education and exercise programme. Four stages–create/update short term goals, complete knowledge test of COPD and exercising safely, reading and watching videos on COPD topics (such as inhalers and healthy eating), completing exercise program and recording walking and symptoms. 11 week program.	1	(1) Programme content was well received, and gains reported included improved activity levels, exercise intensity, and knowledge of the condition. The program was able to be embedded into daily routines. The importance of motivation and self-discipline when following the program was highlighted. Flexibility of program could help, but also hinder, engagement. Support from healthcare professionals was important for engagement, to obtain health advice and technical support.	Difficulties moving through sections of the website and recording completion of sections. The tone of the automated messages caused frustrations The flexibility and the ease the programme could be incorporated into daily living helped participants engage. Participants also liked that their programme was continually monitored and there were interactions with healthcare professionals
Drummond et al. (2016) [15]; France Systematic review	Studies investigating serious games in asthma education for children were included (12 articles included in the review)	Studies examining the impact of Serious Games–“An interactive computer application, with or without a significant hardware component, which has a challenging goal; is fun to play and/or engaging; incorporates some concepts of scoring; and imparts to the user a skill, knowledge, or attitude that can be applied in the real world” (as defined by Bergeron, 2006, p. 17 [20]). Asthma education included all the interventions with the following goals: raising awareness that asthma is a serious chronic disease, ensuring the recognition of the triggers and symptoms of asthma, and/or ensuring effective management of asthma	1, 2, 3, 4	(1) Seven studies reported that serious games were highly favourable for satisfaction. More than 90% of children who played enjoyed it. For some serious games, children reported them as being more fun that their favourite computer game. (2) Mixed results on improving self-efficacy for both children and parents. Knowledge improved pre/post for nearly all studies. Mixed results when other methods of education were compared for changes in asthma knowledge. No change in parent knowledge was observed. (3) When behaviour related to asthma management was measured, children and parents both significantly improved in three of the five studies. (4) No significant difference in acute visits to A&E or hospital admissions. No significant difference in patient symptoms. No significant difference in lung function.	Not specifically reported
Hester et al. (2020) [6]; United Kingdom Randomised controlled trial feasibility study	Feasibility trial comparing usual care versus a novel patient information resource in adults with bronchiectasis 62 adults (24 males: 38 females) were randomised into the control (30) or intervention group (32)	Provided with password protected access to a website with information and videos (as well as a booklet about the website)	1, 4	12 Weeks after accessing the website. (1) 93% (25/27) reported the information useful, particularly the video content. More than 80% thought it was easy to use the website, covered the topics they wanted, and that the right amount of information was given. 64% (18/28) felt knowledge improved. 64% felt they were more able to manage their bronchiectasis. 56% reported partner/family/friend used the information. In the focus group, (n = 11, 8 patients 3 carers), the key themes were learnt new things and a good resource as both patient and carer centred. The information was always available and could not be misplaced. The videos were valued. (4) Both groups improved in HRQoL with no significant differences between the groups.	Found challenges getting onto the internet site. Password meant unable to access for some. Site user experience was not found great by all. Some could not get internet access on computer and needed printed booklet. Not everyone found family members used the platform. 84% of people reported using the information provided. Google analytics showed there were 6456 users with over 20,000 attempted page views. Pages about diet and lifestyle, prognosis and getting a diagnosis, why have I got bronchiectasis, symptoms, who might be seen were most popular.
Houchen-Wolloff et al. (2021) [21]; United Kingdom Nonrandomised feasibility study	100 adults with a confirmed acute exacerbation of COPD (55 males: 45 females) access to a web-based program (SPACE for COPD) for individuals hospitalised with a COPD exacerbation	Website access–comprehensive 11 week online package of exercise and self-management education. Four stages– create and update short term goals, complete knowledge test of COPD and exercising safely, reading and watching videos on COPD topics (such as inhalers and healthy eating), completing exercise program and recording walking and symptoms.	1, 2	(1) Incited internal motivations after exacerbation. Program offered opportunity to learn how to manage condition. 42 completed BCKQ at 6 months. 14 interviews completed, three key themes preparing for, engaging with and benefits of the web-based program. Home-based program of learning and exercise was appealing. External factors, such as reassurance from healthcare professionals, were needed for motivation by some. Patients felt their computer literacy skills were vital to their ability to engage. Navigating the program was complex; some felt it was too steep a learning curve to access the website and disengaged entirely. Family members and friends were a support for those who found use difficult. The exercise reminders to email addresses were met with varied appreciation. Mood, exercise, and practical skills were best learning. The program gave focus to people around their illness and inspired them to challenge and change their health behaviours. (2) Change in BCKQ score was 7.8 (SD 10.2), an increase of 21%, at 6 months following access to the website.	Age or generation felt like a barrier for some people due to their ability. Many lacked confidence in their computer skills or had difficulty engaging
Huang et al. (2021) [7]; China Randomised controlled trial	106 stable COPD Adults patients (68 males: 38 females) enrolled into control (n = 51) or observational group (n = 55)	Intervention consisted of internet-based self-management mode. Using WeChat and a COPD area of the hospital website. Hospital website provided news. WeChat was uploaded by a nurse with COPD knowledge and rehabilitation instructions. Included pictures, popular texts, videos, and images. Patients were requested to reply to the messages confirming whether they understood the information and implemented the information. If not understood by greater than 20% of people in the chat channel, further information would be provided by the nurse. Patients were encouraged to communicate in the group. Where a patient was not engaging, a nurse would make contact. Control group patients had a traditional health education model including dietary/exercise/medication guidance, and oxygen therapy at home.	1, 2, 4	(1) The observational WeChat group were more satisfied with their care (satisfaction rate of 98% versus 84%, X^2^ = 4.887, *p* = 0.027) (2) Self-management ability (measured by daily life management, symptom management, emotional management, information management, and self-efficacy) was significantly greater in the observational WeChat group than control group given a booklet at home (*p* < 0.05). (4) Significantly improved PFT and 6MWD was observed in the intervention group (*p < 0.05*). Quality of life was significantly greater in the WeChat group (*p < 0.05*).	
Jung et al. (2020) [16]; United Kingdom Observational mixed-methods study	10 COPD (MRC 4 or 5) adults were recruited (6 male: 4 female))	8 week program of pulmonary rehabilitation at home using a VR headset. The app was divided into two subgroups: education and rehabilitation. The education section contained high-definition videos to increase retention of patients in completing PR in VR. The rehabilitation section contained physical exercises led by a virtual instructor in the form of a 3D avatar. The final module is a summary of the PR in VR program.	1, 4	Focus groups and interviews were completed. (1) Themes following completion–physical improvements (strength, mobility, and flexibility), improved psychological well-being (mentally relaxed, motivation to get going when I feel depressed), improved HRQoL (performing daily activities was more enjoyable, felt happier spending more time with family and friends), increased confidence (with daily activities, socialising and managing breathlessness), increased feelings of security (knowing healthcare support was there if an exacerbation occurred), effective immersive learning (reported that they learnt things they had never seen before) and patient satisfaction. 5 self-reported surveys were completed before and after use of the VR (4) CRQ-improvements across all domains. Short physical performance battery—improvement post. Patient activation measure—improvement. Edmonton frail scale—improvement. Patient health questionnaire-9—improvement. Generalized anxiety disorder-7—improvement. No statistical analysis undertaken	Technical issues where the camera movement was delayed at times (improved graphics would overcome), suggested a fast-forward, pause, and rewind function would assist with learning as more control over the program. Headset was heavy, although easy to use. Patients reported they had difficult attending rehabilitation centres, and this made it easy. Patients “looked forward” to completing the experience. The use of avatars in the VR made it more personable than booklets.
Kooij et al. (2021) [12]; The Netherlands Observational feasibility study	39 adults with a diagnosis of COPD and recruited during a hospital admission for a COPD exacerbation (9 males: 30 females)	8 week COPD self-management program. Provided with a tablet that had an application installed. The application had an information overview page, a contacts page, the “Lung Attack Action Plan”, 5 information modules (what is the app, what is COPD, physical activity, nutrition, and advantages of ceasing smoking), and questionnaires for monitoring (HADS and Clinical COPD Questionnaire). Participants also had a follow-up session at 4 weeks and 8 weeks after discharge.	1, 2	Evaluation was for 20 weeks. (1) Overall satisfaction rated as 7.7 (SD 1.7) at 8 weeks and 7.0 (SD 2.4) at 20 weeks (out of 10: very satisfied). 93% (26/28) found the application easy to use. The Lung Attack Action Plan was reported by over 90% to be easy to find and 67% (18/27) found it useful. 93% (27/29) found the information presented in modules easy to understand. 33% (9/27) found there was too much information. 66% (19/29) found that video consultations with HCPs saved time; 78% (18/23) were satisfied with the video consults. (2) Knowledge and coping significantly increased over time (*p* = 0.04). No significant change in recognition of symptoms or symptom management (*p* = 0.14).	58% (21/39) of participants expected support with smartphone/tablet use. 19% (7/39) reported their smartphone/tablet use skills to be bad/very bad. Only 36% (13/39) reported tablet use to be very good or good. App use (n = 39) declines over time- week 1 = 100%, week 2 = 85%, week 3 = 82%, week 4–8 = 79%.
Liu et al. (2013) [22]; China Randomised controlled trial	57 adults with COPD and dyspnea (44 males: 13 females) were randomised to an experimental (n = 29) or control group (n = 28)	Intervention was an online breathing program for patients with dyspnea incorporated into a health program for COPD. The program had 4 stages of diagrammatic breathing exercises, each lasting one month. The control group were instructed on the importance of exercise in the same way face to face with handouts. Patients were provided with a username and password access webpage, where there were 4 modules on breathing exercises. Module 1 focussed on pursed lips breathing. Module 2 focusses on deep inspiration- slow expiration. Module 3 focussed on deep inspiration-hold-slow expiration. Module 4 was global exercise (calm breathing then raising arms and lower arms in differing ways while deep breathing)	4	Evaluation at 4 months. (4) The intervention group reported significant improvements in lung function, 6MWT, and quality of life (SGRQ) across all domains compared to control group (*p* < 0.05).	Significantly greater patient adherence with regularly completing the home program was found in the online group than the control group (89% versus 50%)
Marklund et al. (2021) [9]; Sweden Exploratory qualitative study (part of a process evaluation in a parallel group controlled pragmatic pilot trial)	16 adults with COPD (4 males: 12 females) who were allocated to the intervention group and had access to an eHealth tool–the COPD web	Participants were provided with a username and password to access “The COPD Web”. An interactive webpage that was co-created with users. Two sections–one for HCPs and one for patients. Content includes videos, written information, images, and helpful links to other sites. Aim of the site is to increase self-management through knowledge about COPD and strategies to improve health (physical activity level and exercise, breathing techniques, observing symptoms of exacerbations, and advice about making everyday activities less strenuous). Section for registering daily step count (participants provided with pedometer to measure). “News” was emailed to participants.	1	Evaluated at 3 months and 12 months through interviews. (1) 6 people were users, and 10 people were seldom or never users. Users were IT comfortable with a positive view of using the computer. They found the webpage was a carrot to learn more and by know-how they had hope with new insights about self-care. The website affirmed their knowledge, and they were starting to see benefits at 3 months. At 12 months the users found more impact with more time. They had confidence in the expertise of one’s own body. They enjoyed being reminded of various exercises and found the breathing exercises as a way to calm down. All users found that shame and guilt (of smoking) influenced their use of the platform. This was felt to be a source of stress and uncomfortableness that brought them to the site initially. Knowledge about COPD and self-management was a curiosity. Levels of eHealth accessibility were good for most. They felt positive about the COPD Web. Nonusers found the information “scary” and used health status as a motivator when well and excuse when unwell. Nonusers felt the tool was irrelevant, not prioritising using the COPD Web.	Use—38% (6/16) were considered users. Nonusers were not IT comfortable
Morrison et al. (2016) [10]; United Kingdom; Feasibility phase 3 randomised controlled trial	51 Adults with physician-diagnosed symptomatic asthma (13 male: 38 female) 25 participants in the intervention group.	Website access “Living well with asthma” versus usual care website designed to provide understanding and assess current level of asthma control, support optimal medication management, challenge attitudes and concerns around medication, and prompt use of personal action plan.	4	(4) No significant difference in ACQ score or mini-AQLQ scores overall. Activity limitation domain on mini-AQLQ significantly improved. Significant improvement in PAM scores for intervention group compared to control. No significant difference in hospital/A&E visits. No significant difference in routine or nonroutine GP/nurse visits. No significant difference in oral prednisolone course. Significantly fewer reliever puffs taken per average week in education group.	Barriers to accessing the website included available time and opportunity rather than content.
North et al. (2020) [13]; United Kingdom Randomised controlled feasibility trial	41 adults with COPD (post exacerbation with hospital admission) randomised to usual treatment (21) or MyCOPD app (20) (24 male: 17 females.	myCOPD Application consists of education programmes, 6-week online PR, inhaler technique videos, and environmental alerts of weather and pollution. Weekly usage by participants was on average 4.9 days. 75% of participants used the app for more than a week. Were given access for 12 weeks and monitored.	2, 4	(2) Inhaler technique errors at 90 days was significantly lower in myCOPD group (1.2 versus 4) with an adjusted incidence rate ratio of 0.38 (95% CI 0.18–0.8, n = 35). (4) Readmission rate significantly lower in myCOPD group (0.24 versus 0.39, 95% CI mean difference 0.07–1.99). Number of exacerbations significantly less in myCOPD group (1.06 versus 1.88; 95% CI mean difference 0.3–1.07). CAT Score, MRCD, PAM Score, HADS Score, SGRQ Score, WPAI and VSAQ Score no difference between the groups.	Not specifically reported Usage was highest in week one (85% of people accessing daily) and decreased to 40–45% people accessing daily in weeks 9–12.
Park et al. (2020) [14]; Korea Randomised controlled trial	42 adults with COPD (Gold stage 1–3) Adults–42 (79% Males: 11% females)	The intervention group received the smartphone app-based self-management program (SASMP) Patients were provided with the smartphone app for COPD self-management. The app was created applying Bandura Social Cognitive Theory principles. Patients were asked to set achievable goals and were taught strategies to relieve their symptoms. Group texting connected people for peer-to-peer support. Self-monitoring of symptoms was encouraged, with strategies to manage symptoms provided. Group education and exercise were provided to both groups of participants as well.	1, 3, 4	Evaluated at 6 months. (1) Satisfaction with the program was not significantly different between the groups, with a score of 94.55 ± 9.63 versus 89.50 ± 10.50 for the control group. Support for disease management, symptom management, and increasing physical activity level were all scored >90/100. 30% of participants appreciated the support of peers and HCPs through the app. Just over half of the people reported that they learned more about their disease, the importance of exercise, balanced nutrition and increasing physical activity levels, increased understanding of symptom management and level of self-care. (3) No significant difference between the groups in exercise behaviour (min per week). Significantly greater physical activity (total activity and lower sedentary activity % time) in the group with the smartphone app. Significantly greater moderate to vigorous activity in the app group (measured with accelerometer). Self-care behaviour was significantly better in the app group. (4) No significant difference between the groups in exercise capacity, symptoms, self-efficacy, perception of control, or social support.	Recording exercise and symptoms in the app were felt to be a burden by some. Not provided
Robinson et al. (2021) [11]; United States Randomised controlled trial	153 Adults with COPD (142 male; 11 female) were recruited to the trial. Intervention group participants were mailed detailed instructions about the study website. Both groups received an educational booklet and verbal encouragement (75 intervention; 78 control group)	The intervention group were provided with a pedometer and access to a website that contained content to promote physical activity: walking assessment and feedback, individualised step goals, educational tips and motivational message, and an online community (discussion boards)	1, 2, 3, 4	Evaluation took place at 6 months (1) 73% (44/60) response to survey on experience. 95% (42/60) would recommend to others. 75% (33/40) felt the experience helped them stick to their walking. 95% (42/60) reported they would continue to exercise after the research program. 55% (24/44) found educational tips and motivational messages easy to understand—the remaining 45% (20/44) did not use these modules. 25% (11/44) felt they learned helpful information from the online discussion boards. 75% (33/44 reported not using the discussion boards. (2) No significant difference in COPD knowledge. (3) Significantly greater mean daily step count of 1312 steps/day in intervention group (95% CI 600–2024, *p* < 0.001). (4) No significant difference between the groups in 6MWD (mean difference of −12 m, *p* = 0.189). No significant difference in SGRQ, MRCD, MOS-SS, number of acute exacerbations, or number of COPD-related admissions.	Not specifically reported

* Kirkpatrick’s model of evaluation of learning and education, 4 key levels of impact for participants user satisfaction with the technology-enabled patient education (level 1), gain of skills and knowledge (level 2), behavioural change (level 3), and improved health outcomes (level 4) [19] 6MWD = Six minute walk distance; A&E = accident and emergency (ward of hospital); ACQ = asthma control questionnaire; mini-AQLQ = asthma quality-of-life questionnaire; BCKQ = Bristol COPD knowledge questionnaire; CAT = the COPD assessment test; COPD = chronic obstructive pulmonary disease; CRQ = chronic respiratory questionnaire; HAD = hospital anxiety and depression scale; HRQoL = health-related quality of life; MOS-SS = medical outcomes study–social support survey; MRCD = medical research council dyspnea scale; PAM = physical activity monitor; PFT = pulmonary function test; SGRQ = St. George respiratory questionnaire; VSAQ = veterans specific activity questionnaire; WPAI = work productivity and activity questionnaire.

### 2.1. User Satisfaction with Technology-Enabled Patient Education 

For all studies that measured patient satisfaction, technology-enabled patient education was a positive experience by the majority of participants. The majority of participants would recommend the technology-enabled patient education to peers, finding the web-based and application-based education kept them motivated, was easy to use, and was relevant and perceived their knowledge and skills to have improved [5,6,9,12,14,15,16,21,23]. Participants also found that the technology solution meant the information was always readily available and not able to be misplaced, and this was very helpful [6]. Programme content was well received for all approaches, with participants providing feedback that the symptom management, exercise/physical activity, inhaler, and healthy diet learning was most useful across studies [14]. Where technology was used to synchronously connect with healthcare professionals, most participants found this to save time and a welcomed approach [12]. Discussion forums with peers were met with varied enthusiasm, with some studies reporting patients contacted peers readily [7] and other authors stating 75% of participants were not interested [11]. Although, when there was a comparison educational experience not using technology, the satisfaction score was not greater in the technology-enabled model [14]. 

### 2.2. Gain of Skills and Knowledge

A significant increase in measuring learning outcomes has occurred in the last decade with 6 of the 13 studies evaluating gain of skills, knowledge, and attitudes. In adults, technology-enabled patient education showed an increased disease specific knowledge [12,21] and inhaler technique improved [13]. The perception of coping with the condition also increased [12]. Although not all studies found increases, Robinson et al. (2021) reported no change in COPD knowledge [11]. The review by Drummond et al. (2016) showed mixed results in the acquisition of asthma knowledge when comparing the use of computer games for children with other educational modes, but all studies examined found an improvement pre-post [15]. 

### 2.3. Behavioural Change

Self-care, medication adherence, and physical activity levels were the three key behaviour changes that were found to improve significantly following technology-enabled patient education for people with chronic respiratory conditions [11,14,15]. Because only three studies examined behaviour change following technology-enabled patient education for people with chronic respiratory conditions, confidence in effect is limited. This is an area that requires further investigation to understand how learning translates to changed behaviour and then in turn improved health outcomes. Further, no studies examined the maintenance of behaviour longer term. All measures were pre-post intervention, with limited understanding of whether people continued to access the technology-enabled education to remind themselves and maintain their health behaviours in the longer term. 

### 2.4. Improved Health Outcomes 

As has been observed in studies of face-to-face patient education for chronic respiratory diseases [24,25], the impact of technology-enabled patient education on health outcomes is variable. Pulmonary function, quality of life, exercise capacity, anxiety and depression, and healthcare use were examined across the studies. Many studies found significantly improved health-related quality of life, suggesting that technology-enabled patient education for people with chronic respiratory disease has an impact [6,7,8,10,16] Healthcare use, measured as prescribed medication use, unplanned physician/nurse visits, emergency hospital presentations, and hospital admissions, was also found to be significantly lower following the participants involvement in technology-enabled patient education in two studies [10,13] but not in the other two studies that measured hospitalisations [15,23]. A proportion of studies found a significant difference in lung function, exercise capacity, or symptoms [11,13,14,15]. These findings align with aforementioned systematic reviews of face-to-face disease specific chronic respiratory education [24,25], although, a meta-analysis of technology-enabled patient education studies with a rigorous systematic review is needed to confirm this finding. Technology-enabled patient educational programs with greater interactive activities, where the participant uploaded information, appeared to have significant positive impacts on health outcomes—more so than those focussed on information delivery.

### 2.5. Links between Levels of Impact

Two of the included papers have examined all four levels of impact, allowing the examination of the inter-relatedness of patient experience to learning attainment to behaviour change and finally changes in health outcomes [11,15]. Although the study by Drummond et al. [15] was a systematic review, not all studies included looked at all levels of impact. The study by Robinson et al. [23] demonstrated that the positive patient experience did not specifically lead to increased knowledge in COPD when compared to usual care (knowledge increased in both groups). The intervention group did change their behaviours, with greater daily step counts, but this did not lead to significant differences in the health outcomes including exercise capacity (six-minute walk distance), quality of life (St. George respiratory questionnaire), dyspnea (medical research council dyspnea scale), and healthcare use (COPD hospital admissions). This study does not demonstrate that by having a good educative experience using a web-based platform, there is greater learning than what occurs for patients in usual care. Comparable improvements in COPD knowledge were observed, yet the technology-enabled patient education group (who were using pedometers to track daily steps) were more active. However, behaviour change in physical activity levels did change health outcomes compared to usual care. These results suggest impacts are not linked, and other factors may be working to change behaviour beyond knowledge acquisition. However, as this is only one study, further research examining all four levels of anticipated impact (positive experience, learning attainment, behaviour change, and improved health outcomes) needs to be undertaken.

## 3. Technology-Enabled Patient Education—The Good, the Bad, and the Next Steps for Improvement

Technology-enabled patient education for people with respiratory disease has grown significantly in the last decade, with 10 of the 13 papers included here published in the last two years (2020–2021). This shift in how we connect with our patients for their education is likely due to the global COVID-19 pandemic and the need to socially distance for public health. Fortunately, the research conducted in the last two years demonstrates that this new mode of education is well received by patients with chronic respiratory disease, with the benefits of flexibility and decreased time burden to travel for face-to-face education clearly highlighted by patients completing the online education programs. All the studies included in this paper provide insights into the mechanisms by which the patient learning experience can be enhanced when using technology, giving healthcare professionals creating technology-enabled patient education directions that can be taken that enhance experience and impact (Table 1). Specifically, our narrative review highlights that education grounded in topics patients consider relevant to them, and activities that encourage the patient to interact with the platform appear to lead to better user experience may increase learning and subsequent behaviour change and improved health outcomes, although this requires further research for confirmation.

Of upmost importance in technology-enabled learning is that the principles of patient education are maintained and adapted to the new format of using technology to support learning. The education needs to be adaptable, having the potential to be tailored to incorporate who the learner is, what their learning preferences and needs are, and what learning outcomes should be achieved [26]. Technology-enabled learning is not simply transferring learning activities performed in person to be on a screen. The relationship developed between healthcare provider, patient, carers, and peers is shaped differently by the digital world. Patients report challenges in expressing themselves and discussing topics with their healthcare providers during telehealth consultations [27]. Health literacy, cultural responsiveness of the resources, and mental health concerns may negatively impact learning and may be exacerbated by the use of digital technology if digital capabilities are insufficient to access and then navigate the platform(s) [28,29]. 

Access to technology should also be considered; the flexibility of the apps and software may also be an issue for those with no or poor internet capabilities, particularly for those on low incomes with limited available internet data. This situation could also arise for regional remote communities lacking infrastructure for internet access. These challenges were reported by multiple studies when patients were asked about using the web-based platforms and smart device applications. Technical issues such as camera movement being delayed at times due to poor graphics and a lack of control of how fast the program progressed were perceived to influence whether the experience was positive for the patient [16]. Passwords and log-in details being required to access the web-platforms were also barriers [6]. Confidence in using computers, smart phones, and tablets influenced uptake for participants was also reported, and this may influence accessibility through avoidance due to stress levels [21]. Indeed nonusers of the technology-enabled patient education were people who felt “scared” to use computers [9]. Many participants had a desire to receive one-to-one support from healthcare professionals in using the online platforms [5,12]. Confidence and capability in using technology may detract from learning about their chronic respiratory disease as they are learning about using computers while simultaneously trying to learn about their respiratory condition. These insights can be used to adapt current approaches to technology-enabled patient education to overcome such barriers and improve equity of access to patient education. The consideration of an “onboarding” experience at the start of a program could support the person to learn how to use the online platform before they start learning about their condition online. User experience in technology is critical to be intuitive and seamless for patients, whereby someone does not need to “learn” about the features but instead instinctively can navigate around the education modules. The consistency of platform design and navigation is a second approach to decreasing the challenge of using technology for learning. Alternatively, healthcare professionals can selectively enrol people in different models of education choosing face-to-face approaches for those who prefer face-to-face and technology for those who have confidence and a desire for flexibility in when and where they access their educative activities. This is seen in some of the participant inclusion and exclusion of the studies reported here.

The added benefits of flexibility in delivery (timing of learning, types of resource, and approach to learning—synchronous or asynchronous) and the obvious role technology has had in keeping people who are isolated for COVID-19 biosecurity reasons connected can outweigh these negatives when appropriate strategies are used. Although as it is important to recognise that technology-enabled patient education is not just about “face-to-face approaches being implemented using a computer/tablet/phone”, healthcare professionals need to know their scope of practice and areas for improvement in design, development, and delivery of technology-enabled patient education. The Social Learning Theory by Albert Bandura in the early 1970s describes that learning is grounded in relationships between people [30]. According to Bandura [30], individuals experiences, interactions with others, and environment factors all influence learning and perception of the world around a person. Through social support that instils expectation, improves self-efficacy, and uses observational learning, behaviour change can be achieved [30]. Relationships are experienced in differing ways when technology is used to connect people and for supporting learning [31], and trust can be diminished in the context of healthcare provision [32]. Indeed, the study by Bourne et al. [25] the tone of automated messages caused frustrations for patients, highlighting that a nonpersonal “computerised” approach to patient education is not well received and may lead to the disengagement or nonadherence with health behaviour change. Patients are likely to be looking for a sense of human connection in learning and healthcare provision. Technology does not exclude this, however, approaches to technology-enabled learning need to foster this. Considering these challenges and the aforementioned barriers for patients, programs of professional development are readily available to healthcare providers looking to upskill in telemedicine approaches and technology-enabled patient education. Further, expertise in technology-enabled learning and teaching can be found both within the healthcare industry and in other industries, such as higher education and corporate organisational learning and development teams. However, as far as we have progressed with the provision of education and care in this new digital way, it should also be considered that for some this is not suitable or accessible; therefore, a wide range of educative options is needed for participants to choose what works for them.

Adjusting patient education to cultural nuances and the context of learning is also critical to patient engagement, and technology-enabled patient education is no exception to this. The studies presented here in this paper were predominately completed in European cultures and do not provide insights into the cultural diversity of participants. None of the studies specifically examined cultural adaptation of the learning platforms for participants. There was also no specific reference to co-creating the learning packages with learners themselves. A possible next direction for technology-enabled patient education could be to apply the experience-based co-design (EBCD) methodology [33]. This methodology for creating educational experiences has five stages. At Stage 1, key stakeholders are engaged, including healthcare professionals, patients, and possibly family/carers depending on who uses the educational materials. Stage 2, synthesis of information and understanding of current capabilities of the patients, learning needs, learning styles, through the existing literature, clinical guidelines, discussions with stakeholders, and resources to then inform Stage 3. Stage 3 is co-creating curriculum and the technology platform with the learners (patients and/or carers and family). Stage 4 is the launch of the technology-enabled patient education, with patients providing feedback and rapid transformation of the technology platform occurring to remove any barriers to experience or limitations to learner occurring. Finally, stage 5 is completed using the RE-AIM framework to assess reach, effectiveness, adoption, implementation, and maintenance of the learning outcomes following widespread implementation of the technology-enabled patient education. This model, for the purpose of evaluating impact, would be implemented in a rigorous randomised controlled trial with another educational mode for comparison. Through co-design with patients as the learners, culturally and linguistically diverse groups have a sense of ownership over the experience, and cultural barriers are minimised, widening the reach and impact for diverse groups of people.

This narrative review of the literature is limited in application as it was not conducted as a systematic review with appropriate methodological processes and meta-analysis of results as possible. Nonetheless, the synthesis of findings across the aforementioned studies provide insights into the effectiveness and impact of technology-enabled patient education for people with chronic respiratory conditions. This review demonstrates that a logical next step would be to complete a rigorous systematic review, using PRIMSA guidelines [34], a GRADE approach [35], and aligned with Kirkpatrick’s framework of evaluating impact of educational activities [19], to confirm clinical practice guidelines around the use of technology in patient education, etc.

## 4. Conclusions

The rapid changes to society seen during the pandemic means that technology will continue to have a role in supporting healthcare providers and patients to connect for the management of chronic respiratory disease. Further investment in understanding mechanisms and optimising technology-enabled patient education for behaviour change and self-management for people with chronic respiratory disease would be well justified. Additionally, the patient education interventions used must align with clinical guidance, being relevant for the context of the patient and the healthcare system in which the patient is engaged. New interventions need to be appropriately reviewed for quality and impact. While we work towards that research and new ways, maintaining the principles of quality education, interactive experiences that facilitate active learning through patient involvement in educational activities, and healthcare professionals connecting with experts in the field of technology-enabled learning for development of education models position us for the best patient outcomes.

## Data Availability

Not applicable.
